# A Foreign Body Granuloma of the Buccal Mucosa Induced by Honeybee Sting

**DOI:** 10.1155/2017/7902978

**Published:** 2017-03-16

**Authors:** Kazuhiko Yamamoto, Yohei Nakayama, Yumiko Matsusue, Miyako Kurihara, Takahiro Yagyuu, Tadaaki Kirita

**Affiliations:** Department of Oral and Maxillofacial Surgery, Nara Medical University, Kashihara, Nara 634-8522, Japan

## Abstract

A foreign body granuloma of the buccal mucosa induced by honeybee sting was reported. The patient was an 82-year-old female who presented with a submucous mass at the right buccal mucosa. The mass was 20 mm in diameter, elastically firm, partly mobile without pain or tenderness, and covered with almost normal mucosa. MR image did not delineate the lesion clearly. Under clinical diagnosis of a benign tumor, the lesion was excised under local anesthesia. The excised lesion was 14 × 11 × 9 mm in size and solid and yellowish in cut surface. Histologically, the lesion consisted of granulomatous tissue with a few narrow, curved, eosinophilic structures compatible with decomposed fragments of a honeybee sting and was diagnosed as a foreign body granuloma, although the patient did not recall being stung.

## 1. Introduction

Diagnosing a submucous mass of the buccal mucosa is a challenge. Clinically, it can be a local inflammatory reaction, a tumor, a cyst, or other conditions. Imaging of the lesion in addition to clinical findings and anamnesis may be helpful for the diagnosis. However, histological examination is inevitably necessary for a definitive diagnosis.

In this report, we presented the rare case of a foreign body granuloma of the buccal mucosa induced by a honeybee sting, although the patient did not recall being stung.

## 2. Case Presentation

**Figure 1:**
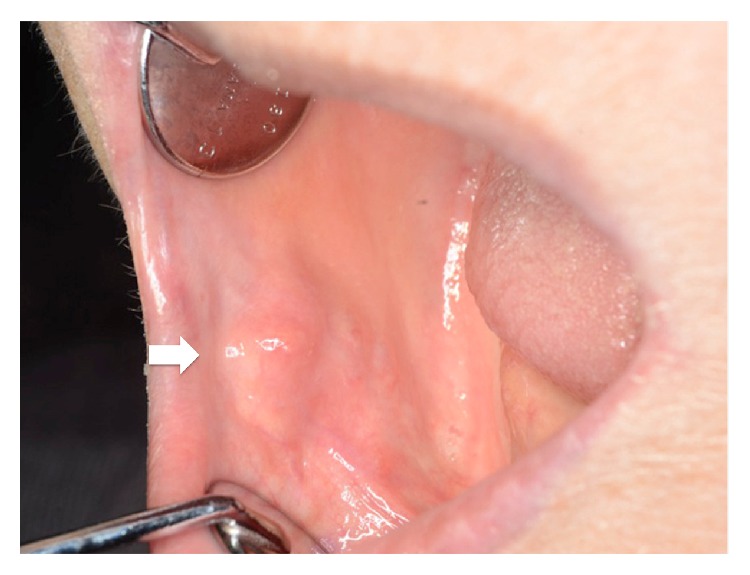
Intraoral finding. A submucous mass at the right buccal mucosa (arrow).

**Figure 2:**
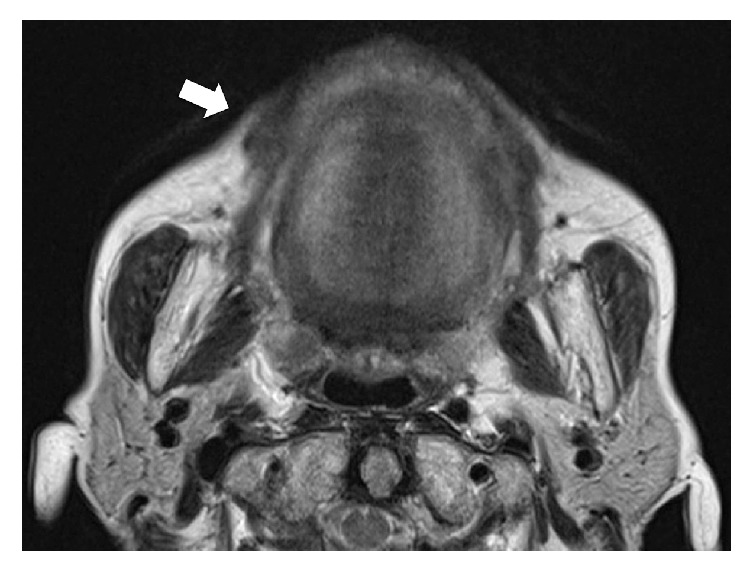
MR image of the lesion (arrow).

**Figure 3:**
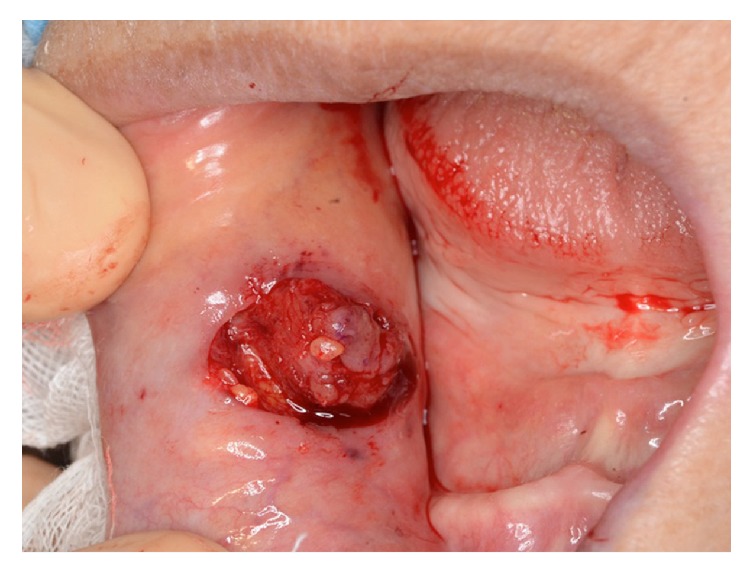
Intraoperative finding.

**Figure 4:**
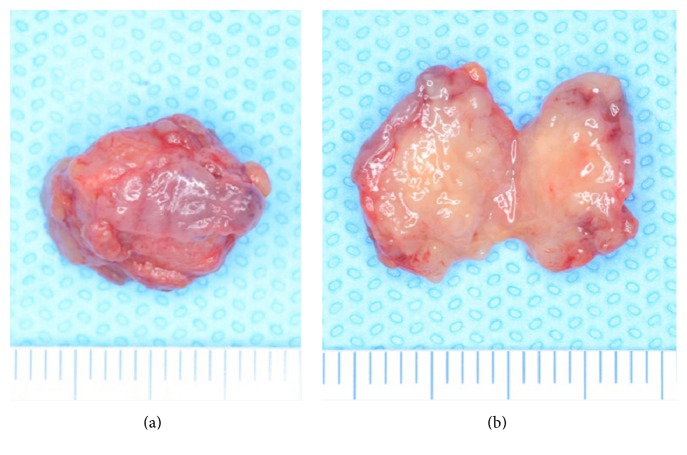
Excised material. (a) Whole material. (b) Cut surface.

**Figure 5:**
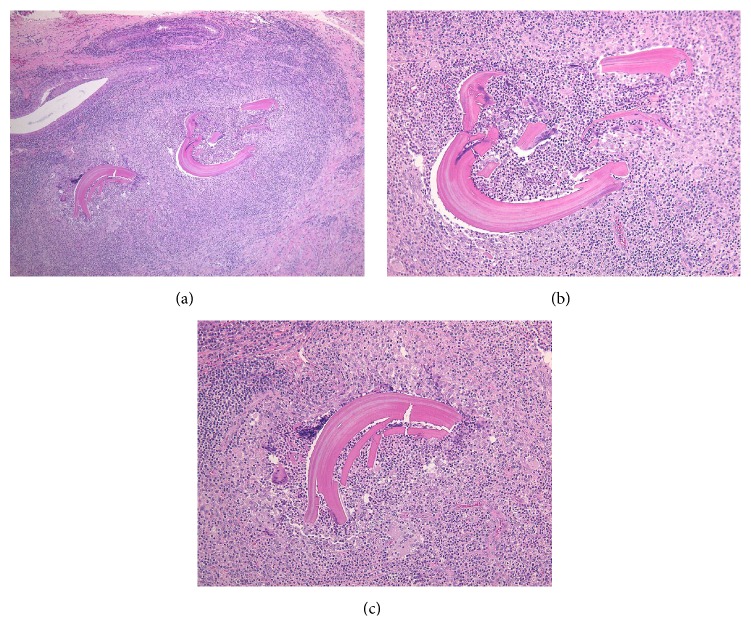
Histological findings (H&E staining). (a) Low-powered view of the lesion. (b, c) High-powered view of narrow, curved, eosinophilic structures.

An 82-year-old female patient was referred to our department for a submucous mass at the right buccal mucosa. The lesion was pointed out at the dental office when she presented for a new prosthesis. The patient had suffered hypertension and senile dementia for several years. The mass was 20 mm in diameter, elastically firm, partly mobile without pain or tenderness (Figure 1). The overlying mucosa was almost normal without fistula. The patient was completely edentulous. The right buccal skin was normal. She said that the mass had been present at least for several months. There had been no episode of injury or surgery at the site according to the patient's memory. MR image did not delineate the lesion clearly (Figure 2). Under the clinical diagnosis of a benign tumor at the buccal mucosa, the lesion was excised under local anesthesia. The mass was easily removed, including a small amount of the healthy surrounding tissue (Figure 3). The overlying mucosa was also excised in a spindle shape. The wound was closed by suture without leaving any dead space. The postoperative course was favorable without tendency of infection or dehiscence of the wound. The excised lesion was 14 × 11 × 9 mm in size (Figure 4(a)) and solid and yellowish in cut surface (Figure 4(b)). Histologically, the lesion consisted of granulomatous tissue with multinuclear giant cells and infiltration of lymphocytes and leukocytes (Figure 5(a)). Minor salivary glands were also observed at the periphery of the lesion. In the granulomatous tissue, a few narrow, curved, eosinophilic structures were observed (Figures 5(b) and 5(c)); therefore, the lesion was diagnosed as a foreign body granuloma. The foreign bodies were considered compatible with decomposed fragments of a honeybee sting from the histological features, although the patient did not recall being stung.

## 3. Discussion

A foreign body granuloma of the oral mucous membrane can be induced by a variety of materials such as teeth, dental materials, oral care goods, tableware, foods, toys, and implants. However, foreign bodies derived from living creatures are rarely found because of the very rare chance for those to enter the oral cavity. A foreign body granuloma developed in the oral mucous membrane after a bee sting is extremely rare and thus is challenging to diagnose. Confirmation of bee stinging is a great help for diagnosis in addition to histological identification of bee sting apparatus.

Bee stings can occur accidentally in daily life. Forestry and field workers who work outdoors are at high risk [1]. Reaction to bee stings varies from a minimal normal reaction to a life-threatening reaction, depending on the volume and toxicity of venom injected, the site of the sting, and the allergic status of the patient [2]. Bee sting typically causes local pain and erythema that resolves within a few hours after the sting. The most critical consequence of bee sting is anaphylactic shock. Bee venom is a complex toxin comprising various kinds of chemicals including melittin, apamin, adolapin, phospholipase A2, hyaluronidase, and histamine [3, 4], and generalized allergic reaction to these chemicals develops in 0.3~5% of people [2]. About 40 deaths per year by bee sting are reported in the United States [2]. In Japan, about 20 people die annually of anaphylaxis caused by Hymenoptera stings [1].

A honeybee sting comprises two barbed lancets and one stylet with a venom canal in its center [5]. The honeybee sting is usually left in the tissue after stinging and is difficult to remove [3]. Therefore, the stinging apparatus together with the venom sac and nerve plexus is left in the tissue and may act as antigen for a long time [6]. The persisting antigens are thought to elicit development of an immune complex-medicated reaction, activating macrophage leading to a granulomatous inflammation [6]. As a result, a foreign body granuloma is formed at the site of the sting [4, 6]. Bee stings can also induce local infection because the stinging apparatus lodged in the tissue is contaminated with pathological bacteria [4].

The honeybee sting apparatus can be identified in the tissue if the sting site was excised shortly after stinging. Histological confirmation of the characteristic structure of honeybee sting apparatus in addition to memory of being stung can lead to a definitive diagnosis. However, the retained materials may be decomposed into small fragments after a long period. Furthermore, the patient might not recall being stung. In such a circumstance, a definitive diagnosis is quite difficult unless the anatomical structure of the sting apparatus is well understood. In the present case, a few narrow, curved, eosinophilic materials were found within the lesion and were decomposed but compatible with the fragments of honeybee sting apparatus. Therefore, the diagnosis of a foreign body granuloma induced by honeybee sting was made.

A foreign body granuloma of the buccal mucosa induced by honeybee stinging is rarely encountered. In the present case, histological identification of the decomposed fragments of a honeybee sting led to the definitive diagnosis, although the patient did not recall being stung.
